# Quantifying iodine concentration in the normal bowel wall using dual-energy CT: influence of patient and contrast characteristics

**DOI:** 10.1038/s41598-023-50238-6

**Published:** 2023-12-19

**Authors:** Majida Nehnahi, Gabriel Simon, Romain Moinet, Gael Piton, Camille Camelin, Maxime Ronot, Éric Delabrousse, Paul Calame

**Affiliations:** 1 Department of Radiology, University of Bourgogne Franche-Comté, CHU Besançon, 25030 Besançon, France; 2 Medical Intensive Care Unit, University of Bourgogne Franche-Comté, CHU Besançon, 25030 Besançon, France; 3 Department of Radiology, University Hospitals Paris Nord Val-de-Seine, AP-HP, Beaujon, 92110 Clichy, France; 4 EA 4662 Nanomedicine Lab, Imagery and Therapeutics, University of Franche-Comté, Besançon, France

**Keywords:** Gastroenterology, Gastrointestinal diseases

## Abstract

This study aimed to establish quantitative references of the normal bowel wall iodine concentration (BWIC) using dual energy CT (DECT). This single-center retrospective study included 248 patients with no history of gastrointestinal disease who underwent abdominal contrast-enhanced DECT between January and April 2022. The BWIC was normalized by the iodine concentration of upper abdominal organs (BWIC_organ,_) and the iodine concentration (IC) of the aorta (BWIC_aorta_). BWIC decreased from the stomach to the rectum (mean 2.16 ± 0.63 vs. 2.19 ± 0.63 vs. 2.1 ± 0.58 vs. 1.67 ± 0.47 vs. 1.31 ± 0.4 vs. 1.18 ± 0.34 vs. 0.94 ± 0.26 mgI/mL for the stomach, duodenum, jejunum, ileum, right colon, left colon and rectum, respectively; *P* < 0.001). By multivariate analysis, BWIC was associated with a higher BMI (OR:1.01, 95% CI 1.00–1.02, *P* < 0.001) and with a higher injected contrast dose (OR: 1.51; 95% CI 1.36–1.66, *P* < 0.001 and 2.06; 95% CI 1.88–2.26, *P* < 0.001 for 500 mgI/kg and 600 mgI/kg doses taking 400 mgI/kg dose as reference). The BWIC_organ_ was shown independent from patients and contrast-related variables while the BWIC_aorta_ was not. BWIC varies according to bowel segments and is dependent on the total iodine dose injected. It shall be normalized with the IC of the upper abdominal organs.

## Introduction

Advancements in computed tomography (CT) technology have revolutionized medical diagnostics, with iodinated contrast agents playing a pivotal role in abnormalities diagnostics^1^. Comparisons of contrast media with varying iodine strengths (milligrams of iodine per milliliter (mgI/mL)), of different contrast doses or administration schemes have been reported in numerous studies^2–4^ to optimize diagnostic accuracy for diverse conditions spanning abdominal, thoracic, and cardiovascular diseases^4–9^. Surprisingly, this approach to dose and protocol optimization has never been conducted specifically for the gut.

A broad spectrum of enhancement abnormalities can be observed in the bowel wall associated with inflammatory conditions, infectious diseases, ischemic events, trauma, and both benign and malignant neoplasms. Combined with morphologic observations, these abnormalities provide helpful information for differential diagnosis^10–14^. However, the assessment of enhancement patterns and degrees is inherently subjective and subject to significant inter-reader variability^13,15^. Using unenhanced images or multiphasic protocols may reduce this variability, but inconsistencies remain^12,15^.

Over the last decade, Dual Energy Computed Tomography (DECT) technology has seen a rise in adoption^16,17^. Several studies have shown that DECT can accurately quantify iodine concentration^18–20^. However, despite the improvements that could be expected from such an accurate measurement, it is not widely used, and there is a scarcity of published data. This underutilization may stem from the absence of standardized methodologies and established reference values for normal bowel wall iodine concentration (BWIC) measurements, hindering its broader implementation in clinical practice.

Thus, this study aimed to use DECT to measure the BWIC in different bowel segments after administering various iodinated contrast agent protocols to provide reference values that could serve as a benchmark for future studies in assessing contrast abnormalities in the different bowel segments.

## Material and methods

### Study population

**Figure 1:**
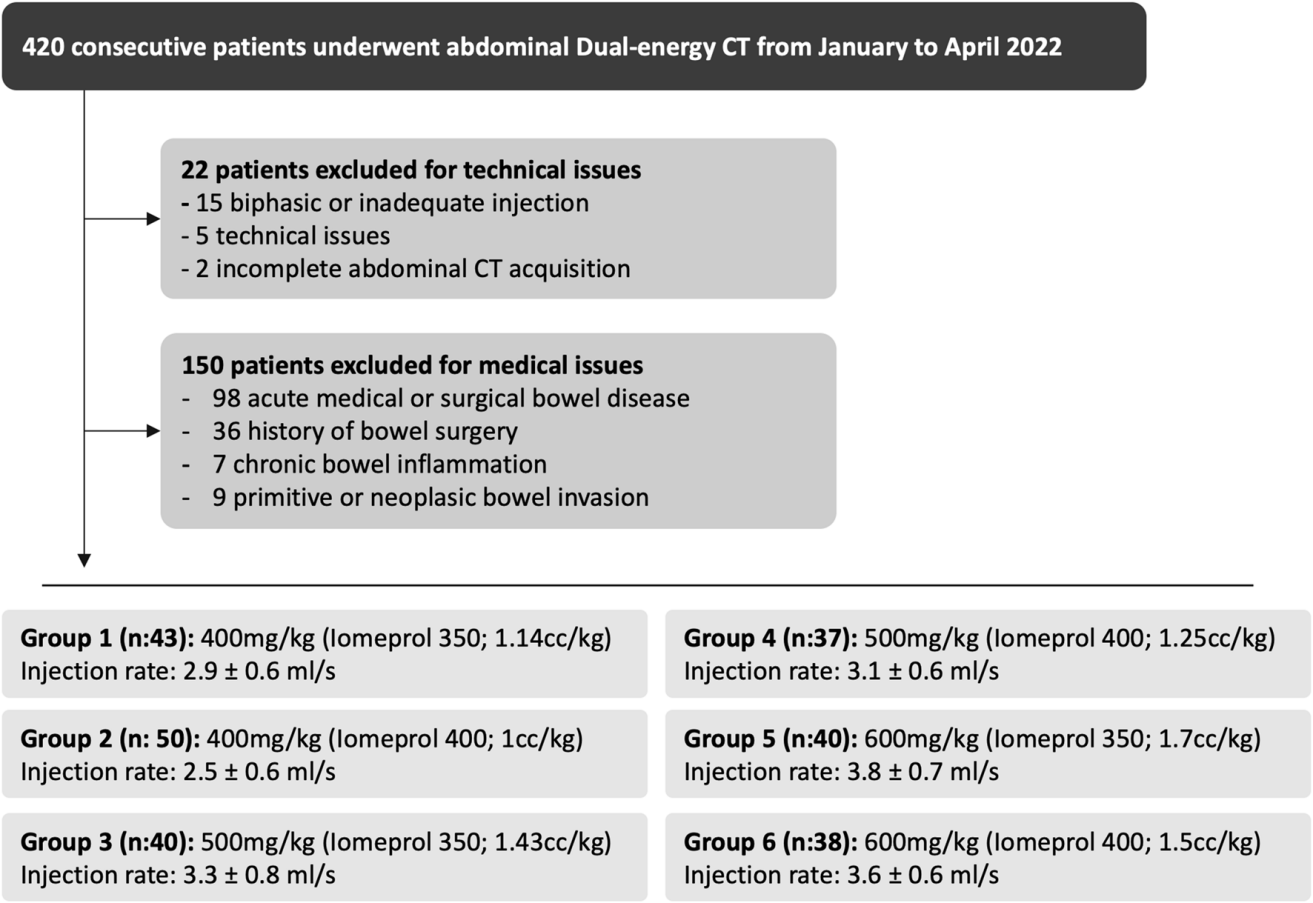
Flow chart of the study population.

This single-center retrospective study was approved by the CERIM (Medical Imaging Research Ethics Committee) IRB (CRM-2212-312) and the need for informed consent was waived. All consecutive patients from January 31st, 2022, to April 22nd, 2022, referred for an abdomen CT, were evaluated for inclusion. Abdominal CT were performed for the following reasons: 130 patients for acute abdominal pain, 67 for hepatobiliary and pancreatic disease, 56 for clinical occlusive syndrome, 52 post-operative control, 41 whole body CT scan for trauma, 22 for neoplasia staging, 20 for acute mesenteric ischemia suspicion, 15 for preoperative vascular assessment and 17 for other reason. Among them, 150 patients with a history of bowel surgery, any ongoing or known history of gastrointestinal disease, pre-surgical assessment of suspected or known structural gastrointestinal disease, and patients requiring evaluation of possible bowel invasion by malignant neoplasms were excluded (Fig. 1).

### Abdominal spectral CT protocol

CT scans were acquired in helical mode with a dual-layer detector allowing conventional and dual-energy images in a single acquisition with a single kVp (IQon Spectral CT, Philips Healthcare, Cleveland, OH, USA). CT scan settings were set with tube voltage 120 kV detector configuration = 64 × 0.625 mm, rotation time 0.4 s, 1.2 pitch and dose modulation with DoseRight Index 14.

### Injection protocol

At our center, the total injected dose of iodine varies according to the indication for abdominal CT, with three increasing doses (400 mgI/kg body weight (BW), 500 mgI/kg, and 600 mgI/kg) using either iomeprol (Iomeron^®^) 350 or iomeprol (Iomeron^®^) 400 administered intravenously with a dual-head power injector at a standard flow rate of 2–5 mL/s with a fixed duration of 30 s. 400 mgI/kg are used for suspected gastrointestinal disease, 500 mgI/kg are used for hepatopancreatic or kidney disease 600 mgI/kg are used for suspected acute mesenteric ischemia, vascular disease, or active bleeding. These different injection protocols enabled us to define six distinct groups for analysis: Two groups received 400 mgI/kg using iomeprol 350 mgI/mL (1 mL/kg) or iomeprol 400 mgI/mL (1.14 mL/kg). Two other groups received 500 mgI/kg using iomeprol 400 mgI/mL (1.25 mL/kg) or iomeprol 350 mgI/mL (1.43 mL/kg). The last two groups received 600 mgI/kg using iomeprol 400 mgI/mL (1.5 mL/kg) and iomeprol 350 mgI/mL (1.7 mL/kg). The portal venous phase was acquired 80 s after initiating the contrast injection. All methods were performed in accordance with the relevant guidelines and regulations.

### Dual-energy post-processing

DECT post-processing was performed using dedicated software (IntelliSpace Portal) with iodine-map, monoenergetic (70-keV), and virtual non-enhanced (VNC) applications. Iodine-map reconstructions (window, 350 HU; level, 60 HU) were predetermined to provide optimal signal-to-noise and contrast-to-noise ratio. Axial iodine-map reconstructions were generated using a slice width of 2.5 mm and an increment of 2.5 mm.

### Quantitative image analysis

**Figure 2:**
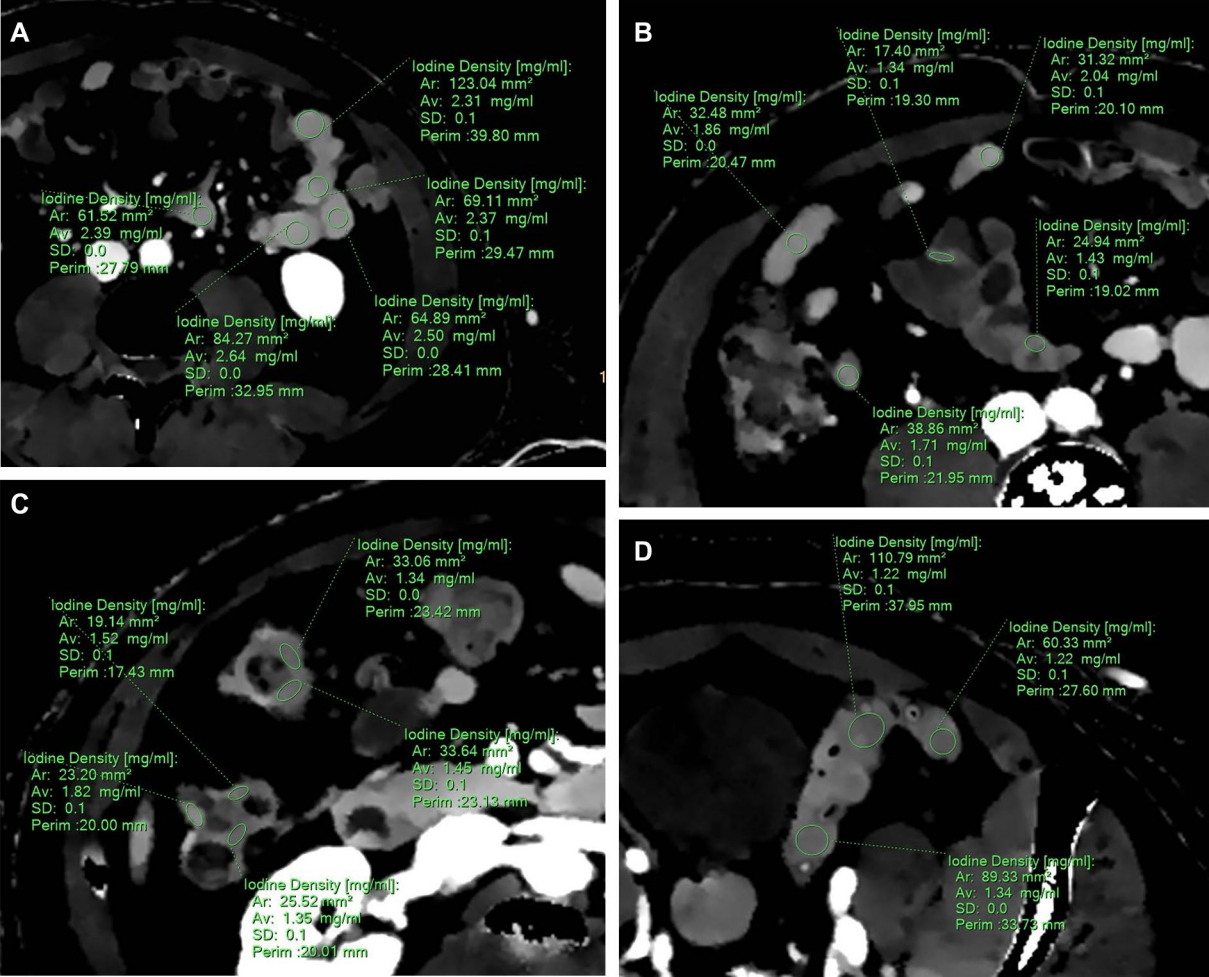
Example of iodine concentration measurement using density iodine map for each bowel segment. (**A**) Five ROI were placed within the jejunum wall. (**B**) Five ROI were placed within the ileum wall. (**C**) Five ROI were placed within the right colon. (**D**) Three ROI was placed within the left colon.

All the individual DECT image sets were retrospectively reviewed by a first radiologist with 4 years of experience in abdominal imaging on a PACS workstation (Carestream Health, Rochester, NY, USA). The reader was blinded to each patient’s contrast dose and iodine strength. Five regions of interest (ROIs) were placed within each bowel segment wall, i.e., within the stomach, the duodenum, the jejunum, the ileum, the right and left colon, and the rectum walls (Fig. 2). ROIs were circular when possible (mainly in the stomach, jejunum, and ileum) or manually drawn (for the right colon, left colon, and rectum from different sections). Jejunum and ileum were differentiated by their location (jejunum in left hypochondrium and ileum in right iliac fossa) and by their appearance (smaller diameter and presence of conivent valves in the wall for the jejunum). To evaluate inter-reader agreement, a second radiologist (5 years of experience in abdominal imaging) assessed the study image sets using the same methodology used by the first reader and measured the iodine concentrations for the first 120 patients enrolled in the study.

### Normalized iodine concentration

The iodine concentration of the bowel wall was normalized by two different approaches:

- Using the iodine concentration of the upper abdominal organs, calculated as the mean of five other ROIs placed in the upper abdominal organs (one ROI drawn in the right liver lobe, one ROI in the left liver lobe, one ROI in the pancreas and two ROIs in the spleen).
- Using the iodine concentration of the aorta calculated using a single ROI within the abdominal aorta

The normalized bowel wall iodine concentration was then calculated by dividing the mean BWIC by the IC of the upper abdominal organs or the aorta IC, resulting in the BWIC_organ_ or the BWIC_aorta_.

### Statistical analysis

Continuous data are expressed as mean ± standard deviation (SD) (for normally distributed data) and categorical variables as numbers (percentage). Continuous variables were compared using the Wilcoxon test (for non-normal distributions verified by the Shapiro–Wilk tests) or the Student t-test when the distribution was normal. Categorical variables were compared with the chi-square or Fisher’s exact test as appropriate.

The interclass correlation coefficient (ICC) was used to assess the correlation between iodine concentration along the different bowel segments between readers 1 and 2 (0.21–0.40, fair; 0.41–0.60, moderate; 0.61–0.80, substantial; 0.81–1.00, almost perfect)^21^. The BWIC was fitted into a generalized linear mixed model. Age, sex, body mass index (BMI), bowel segment, total iodine injected dose, and iodinate concentration of the contrast agent were entered as fixed effect and the subject as a random effect. The same model was used for the BWIC_organ_ and the BWIC_aorta_. ICC were calculated to assess the consistency between the BWIC and the BWIC_organ_, the BWIC_aorta_ and the liver IC. The significance of the comparison between ICCs was assessed using Fisher’s z-test, considering the number of subjects in each group. A *P*-value < 0.05 was considered statistically significant. All analyses were performed with R Foundation for Statistical Computing, Vienna, Austria.

## Results

### Study population

A total of 420 patients underwent an abdominal CT examination from January 31st to April 22nd, 2022, of whom 172 were excluded (Fig. 1), leading to a final cohort of 248 patients who met the inclusion criteria and were included in the study (Fig. 1). The mean age was 57 ± 20 years (range 18–93 years old) with a sex ratio of 1.08 (129 men/119 women). The mean weight was 72 ± 15 kg, the mean height was 167 ± 11 cm, and the mean body mass index (BMI) was 25 ± 4 kg/cm^2^.

The characteristics of the study population according to the six injection protocols are detailed in Table 1*.* Age and sex significantly differed across groups (*P* = 0.001 and *P* = 0.02, respectively). However, no difference was observed between the six groups according to height, weight, or BMI (*P* = 0.233, *P* = 0.203, and *P* = 0.652). A total of 125 (50%) patients received an intravenous injection of Iomeron^®^ 400 (400 mgI/mL) and 123/248 (50) an intravenous injection of Iomeron^®^ 350 (350 mgI/mL). Ninety-three patients (38%) received a contrast dose of 400 mgI/kg, 77/248 (31%) a contrast dose of 500 mgI/kg, and 77/248 (31%) a contrast dose of 600 mgI/kg.

**Table 1:** Characteristics of the study population.

Variables	400 mgI/kg		500 mgI/kg		600 mgI/kg		*P* value*
	iomeprol 350	iomeprol 400	iomeprol 350	iomeprol 400	iomeprol 350	iomeprol 400	
	n = 43	n = 50	n = 40	n = 37	n = 40	n = 38	
Age	62 ± 19	62 ± 20	52 ± 23	52 ± 22	54 ± 19	61 ± 19	0.145
Sex male	19 (44)	17 (34)	24 (60)	16 (43)	28 (70)	16 (42)	0.108
Height, cm	169 ± 9	169 ± 9	166 ± 9	172 ± 9	164 ± 8	167 ± 8.3	0.393
Weight, kg	77 ± 16	74 ± 16	69.5 ± 16.2	72.15 ± 15.95	67.84 ± 16.21	71 ± 11.7	> 0.99
Body mass index	26.2 ± 4	25.2 ± 4	25.3 ± 5	24 ± 4	24 ± 5	25 ± 5	> 0.99
Contrast injection rate	2.9 ± 0.6	2.5 ± 0.6	3.3 ± 0.8	3.1 ± 0.6	3.8 ± 0.7	3.6 ± 0.6	< 0.001

*Comparison of the six different group using ANOVA test, with adjusted *P* values according to the Benjamini–Hochberg correction method for multiple comparisons.

Normal quantitative variables are expressed by mean ± standard deviation.

Numbers in parentheses are percentages.

#### Bowel wall iodine concentration according to the bowel segment

**Figure 3:**
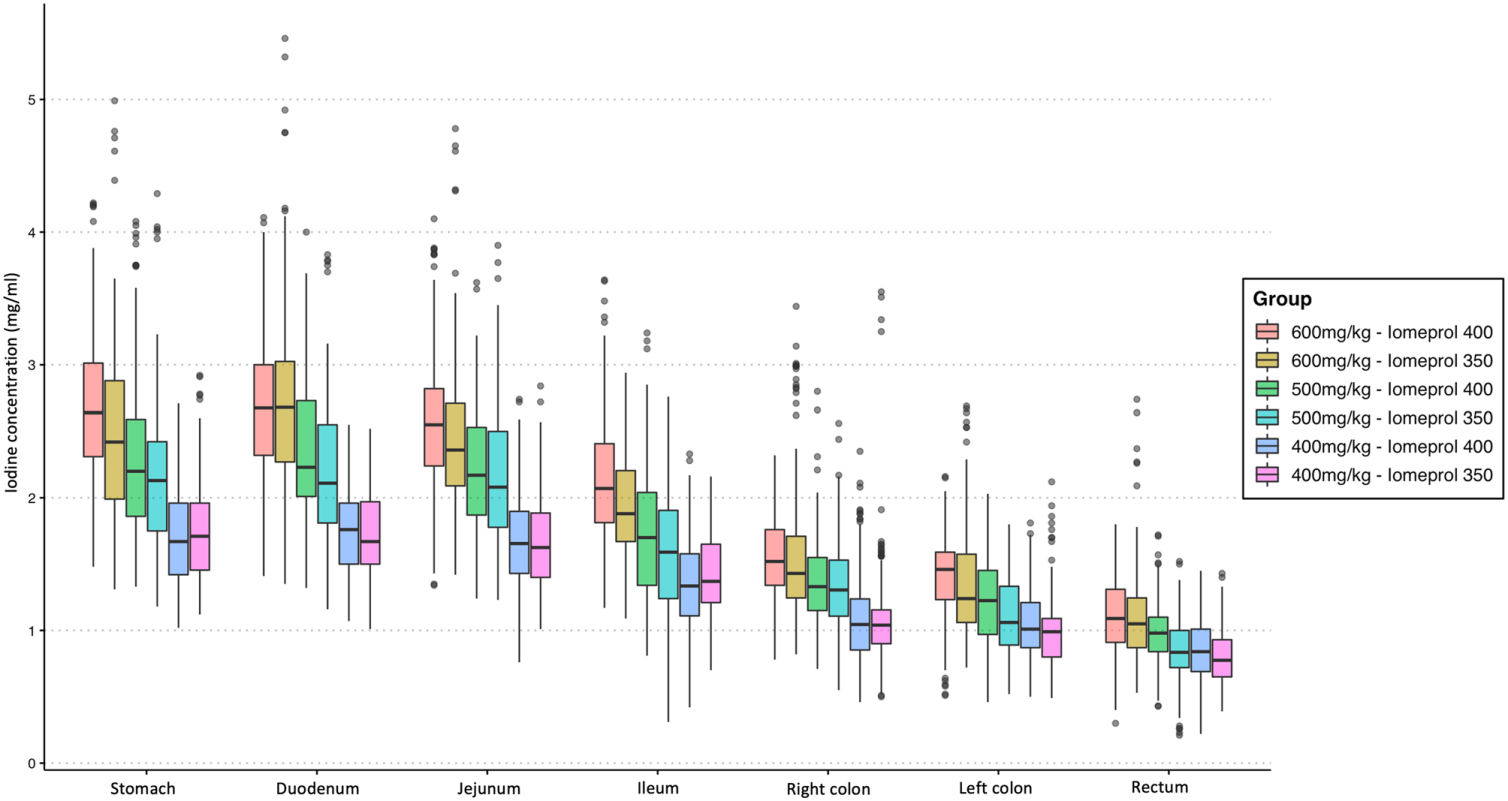
Box plot diagrams illustrating the iodine concentration (mgI/mL) in each group of patients according to each bowel segment.

**Figure 4:**
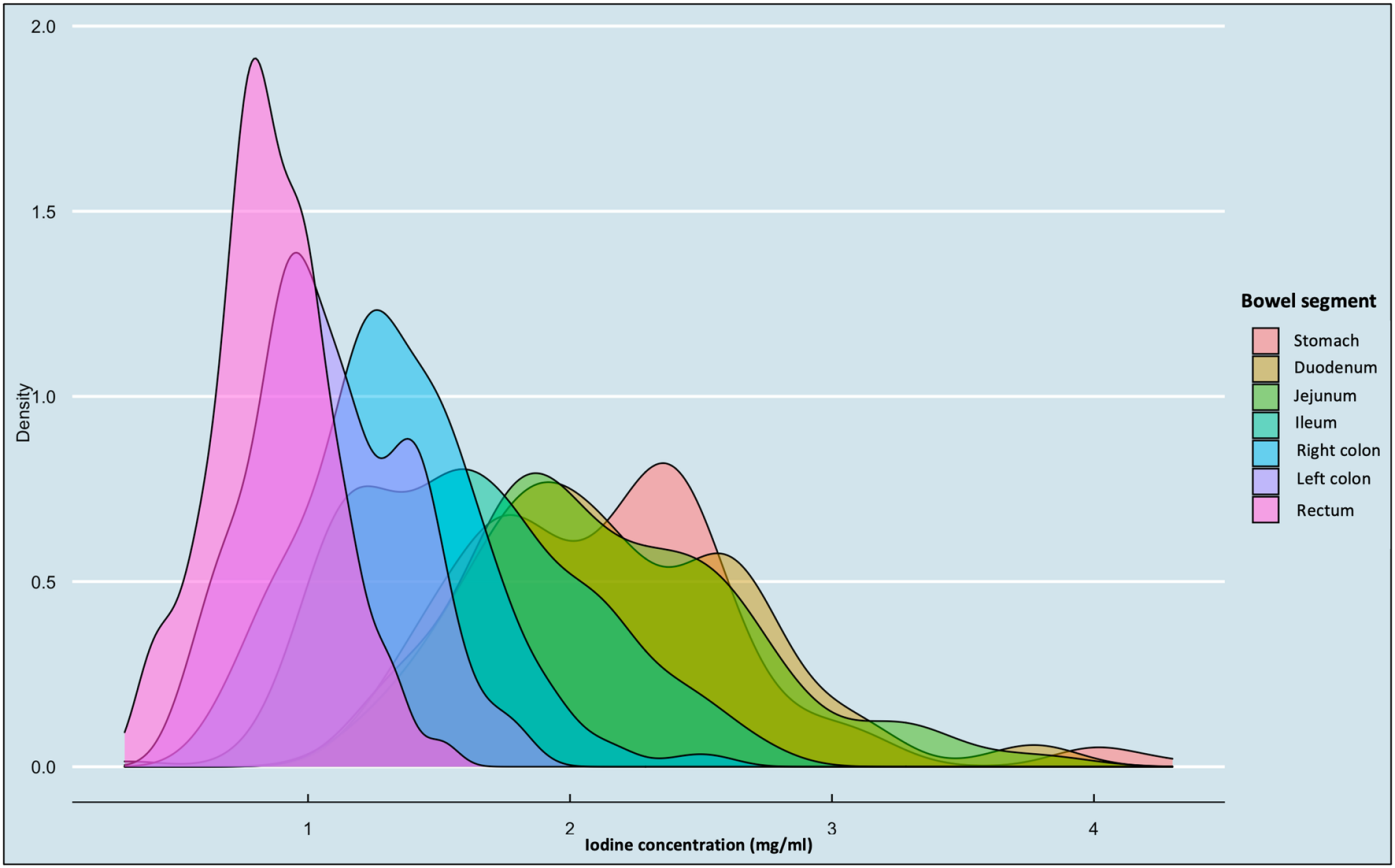
Density histograms of iodine concentration according to each bowel segment in the group 500 mgI/kg using Iomeprol 400 mgI/mL. The Y-line on a density plot represents the cumulative percentage estimate.

Table 2 and Fig. 3 show the results of the measurements of bowel wall iodine concentrations of the stomach and the different segments of the small and large bowels for the six injection protocols. The bowel wall iodine concentration decreased progressively from the stomach to the rectum (mean of 2.16 ± 0.63 vs. 2.19 ± 0.63 vs. 2.1 ± 0.58 vs. 1.67 ± 0.47 vs. 1.31 ± 0.4 vs. 1.18 ± 0.34 vs. 0.94 ± 0.26 mgI/mL for the stomach, duodenum, jejunum, ileum, right colon, left colon and rectum, respectively; *P* < 0.001; Fig. 4).

**Table 2:** Iodine concentration (mgI/mL) measured in the bowel walls according to the bowel segment, contrast medium and dose injected.

	Stomach	Duodenum	jejunum	Ileum	Right colon	Left colon	Rectum	*P* value*
600 mgI/Kg								
Iomeprol 400	2.7 ± 0.56	2.69 ± 0.55	2.57 ± 0.52	2.12 ± 0.46	1.54 ± 0.28	1.43 ± 0.3	1.09 ± 0.24	** < 0.001**
Iomeprol 350	2.5 ± 0.64	2.69 ± 0.67	2.49 ± 0.58	1.93 ± 0.35	1.56 ± 0.5	1.36 ± 0.41	1.10 ± 0.32	** < 0.001**
500 mgI/Kg								
Iomeprol 400	2.33 ± 0.62	2.35 ± 0.5	2.21 ± 0.44	1.72 ± 0.47	1.37 ± 0.31	1.23 ± 0.31	0.98 ± 0.2	**< 0.001**
Iomeprol 350	2.14 ± 0.53	2.18 ± 0.52	2.16 ± 0.52	1.61 ± 0.41	1.33 ± 0.32	1.09 ± 0.27	0.85 ± 0.21	** < 0.001**
400 mgI/Kg								
Iomeprol 400	1.71 ± 0.35	1.74 ± 0.3	1.68 ± 0.35	1.34 ± 0.34	1.09 ± 0.3	1.05 ± 0.26	0.85 ± 0.2	** < 0.001**
Iomeprol 350	1.74 ± 0.35	1.72 ± 0.31	1.66 ± 0.32	1.42 ± 0.24	1.09 ± 0.39	0.99 ± 0.24	0.79 ± 0.18	** < 0.001**

*Comparison of the six different group using ANOVA test, with adjusted *P* values according to the Benjamini–Hochberg correction method for multiple comparisons.

Normal quantitative variables are expressed by mean ± standard deviation.

Significant values are in bold.

Higher wall iodine concentrations were obtained for each bowel segment using higher contrast medium iodine strength and a higher injected contrast dose. Indeed, the highest BWIC were observed with the highest dose (600 mgI/kg) and the highest iodine strength (400 mgI/mL).

#### Inter-reader agreement for iodine concentration measurements

The interclass correlation coefficients were 0.87 (95% confidence interval (CI) 0.87–0.92), 0.85 (95% CI 0.81–0.88), 0.89 (95% CI 0.87–0.92), 0.83 (95% CI 0.78–0.86), 0.58 (95% CI 0.49–0.66), 0.63 (95% CI 0.55–0.70) and 0.57 (95% CI 0.48–0.65) for the stomach, duodenum, jejunum, ileum, right colon, left colon, and rectum BWIC, respectively.

The higher the injected contrast dose, the higher the inter-rater agreement with a interclass coefficient correlation of 0.59 [95% CI 0.54–0.63], 0.70 [95% CI 0.65–0.76], and 0.85 [95% CI 0.79–0.88] with 400 mgI/kg, 500 mgI/kg and 600 mgI/kg, respectively.

#### Multivariate analysis

Table 3 shows the results of the multivariate mixed linear regression analysis. The BWIC was significantly associated with the injected dose of iodine (OR: 1.51; 95% CI 1.36–1.66, *P* < 0.001 and 2.06; 95% CI 1.88–2.26, *P* < 0.001 for 500 mgI/kg and 600 mgI/kg contrast doses taking 400 mgI/kg dose as reference), and with the BMI (OR = 1.01 [95% CI 1.01–1.02], *P* < 0.017). The iodine concentrations were also influenced by the bowel segment (OR = 0.64 [CI 95% 0.61–0.68], 0.45 [CI 95% 0.42–0.47], 0.39 [CI 95% 0.37–0.41], OR = 0.31 [CI 95% 0.29–0.32], all *P* < 0.001 for the ileum, right colon, left colon and rectum, taking the stomach as reference).

**Table 3:** Multivariate linear regression of the factors associated with iodine concentration.

Variables	Model 1			Model 2			Model 3		
	Variables associated with BWIC			Variables associated with BWIC_organ_			Varaibles associated with BWIC_aorta_		
	Odds Ratio	95% Confidence interval	*P* value	Odds Ratio	95% Confidence interval	*P* value	Odds Ratio	95% Confidence interval	*P* value
Age	1	1.00–1.00	0.052	1	0.99–1.00	0.747	1	0.99–1.00	0.063
Male sex	1.04	0.95–1.12	0.395	0.99	0.96–1.02	0.562	0.99	0.98–0.99	**0.043**
Body mass index	1.01	1.00–1.02	**0.008**	1	0.99–1.00	0.371	1.01	0.99–1.00	0.141
Contrast agent									
iomeprol 350	*Ref*	*Ref*	*Ref*	*Ref*	*Ref*	*Ref*	*Ref*	*Ref*	*Ref*
iomeprol 400	1.07	0.99–1.16	0.091	1	0.96–1.03	0.950	1	0.97–1.03	0.306
Iodine concentration									
400 mg iodine/kg	*Ref*	*Ref*	*Ref*	*Ref*	*Ref*	*Ref*	*Ref*	*Ref*	*Ref*
500 mg iodine/kg	1.51	1.36–1.66	** < 0.001**	1	0.96–1.03	0.946	1	0.97–1.03	0.166
600 mg iodine/kg	2.06	1.88–2.27	** < 0.001**	1.03	0.99–1.06	0.130	1.02	0.99–1.05	0.054
Bowel segment									
Stomach	*Ref*	*Ref*	*Ref*	*Ref*	*Ref*	*Ref*	*Ref*	*Ref*	*Ref*
Duodenum	1.06	0.99–1.13	0.053	1.03	1.01–1.05	**0.005**	1.01	1.00–1.03	**0.023**
Jejunum	0.96	0.90–1.02	0.231	0.98	0.96–1.00	0.147	0.99	0.98–1.00	0.108
Ileum	0.64	0.61–0.68	** < 0.001**	0.84	0.82–0.85	** < 0.001**	0.91	0.90–0.92	** < 0.001**
Right colon	0.45	0.42–0.47	** < 0.001**	0.72	0.71–0.73	** < 0.001**	0.84	0.83–0.86	** < 0.001**
Left colon	0.39	0.37–0.41	** < 0.001**	0.69	0.68–0.70	** < 0.001**	0.82	0.81–0.82	** < 0.001**
Rectum	0.31	0.29–0.32	** < 0.001**	0.62	0.61–0.64	** < 0.001**	0.78	0.77–0.80	** < 0.001**

BWIC = Bowel wall iodine concentration; BWIC_organ_: Bowel wall iodine concentration normalized on background upper abdominal organs; BWIC_aorta_: Bowel wall iodine concentration normalized on aorta.

Significant values are in bold.

#### Normalized iodine concentration

**Figure 5:**
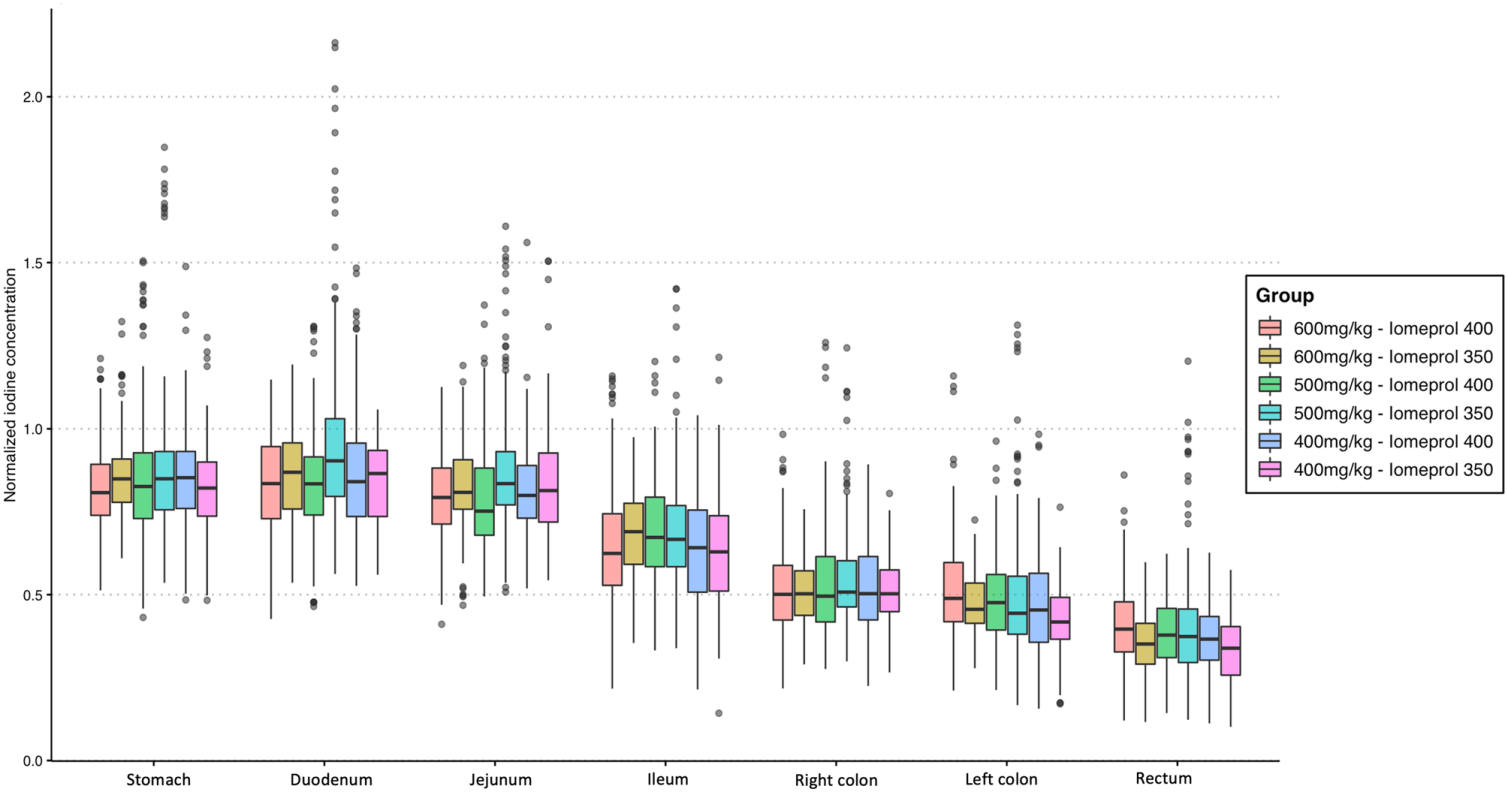
Box plot diagrams of normalization with a background iodine concentration of abdominal organs according to each bowel segment.

The mean iodine concentration of the upper abdominal organs was 2.57 ± 0.63 (range: 1.28, 4.31) mgI/mL and the mean aorta iodine concentration was 4.96 ± 1.32 (range: 2.36, 9.45). After normalization with the iodine concentration of the upper abdominal organs, no significant difference in the normalized bowel wall iodine concentration (BWIC_organ_) was observed according to the iodine strength or the injected dose (Table 3, Fig. 5). However, differences remained after normalization with the aorta (BWIC_aorta_) (Table 3). 600 mgI/kg exhibited an OR of 1.02 (CI 95% 0.99–1.05, *P* = 0.054), male sex an OR of 0.99 (CI 95% 0.98–0.99, *P* = 0.043) and age an OR of 1 (CI 95% 0.99–1, *P* = 0.063). Moreover, the interclass coefficient correlation between the BWIC and iodine concentration of the upper abdominal organs was higher than with the iodine concentration of the aorta (ICC = 0.64 CI 95% [0.61–0.67] vs. 0.53 CI 95% [0.487–0.57], *P* < 0.001. Therefore, normalization with the iodine concentration of the upper abdominal organs (BWIC_organ_) was used for the rest of the analyses.

BWIC_organ_ for each bowel segment. It was 0.85 ± 0.16 (range: 0.46, 1.76); 0.87 ± 0.18 (range: 0.49, 2.04); 0.82 ± 0.15 (range: 0.5, 1.53); 0.66 ± 0.15 (range of 0.29, 1.34); 0.52 ± 0.12 (range of 0.25, 1.12); 0.47 ± 0.14 (range: 0.17, 1.26) and 0.38 ± 0.11 (range: 0.12, 0.96) for the stomach, duodenum, jejunum, ileum, right colon, left colon and rectum, respectively.

## Discussion

Our study evaluated various bowel segments’ bowel wall iodine concentration at the portal venous phase according to the total contrast injected dose and the contrast medium strength. We observed that the BWIC decreased from the stomach to the rectum. We also showed a BWIC increase with the dose of iodine injected (with 600 mgI/kg > 500 mgI/kg > 400 mgI/kg). However, after normalizing the iodine concentration to the background uptake of abdominal organs, the progressive decrease in iodine concentration from the stomach towards the rectum was maintained, but the influence of the dose of iodine injected was not.

Several studies have attempted to quantify the iodine concentrations in pathological processes of different organs (e.g., pancreas, liver, gut) by comparing them to healthy subjects^11,22–24^. In the bowel, Lourenco et al.^24^ compared the iodine concentrations between healthy and ischemic bowel walls in patients suspected of acute mesenteric ischemia and reported significant differences between the groups. De Kock et al.^11^ compared the bowel wall iodine concentration between healthy subjects and patients with Crohn’s disease. In the cohort of healthy subjects, the iodine concentration in the ileum wall was lower than the concentrations found in our study, which the use of a different contrast medium can explain. Together, these studies show that the evaluation of bowel enhancement requires standardization.

Our results contribute to this process. Firstly, we observed a decrease in iodine concentration from the stomach to the right colon, which likely correlates with the reduction in vascularization and function along the gut. Therefore, analyses should not be performed for the entire bowel but should consider each segment. We showed a progressive decrease in the wall iodine concentration from the stomach to the left colon. Therefore, the range of normal IC value differs for each gut segment, with direct consequences for the interpretation of images. For instance, the value of iodine concentration that defined a colonic enhancement anomaly cannot be the same values as the duodenal or jejunum. Our results may serve as benchmark for future studies.

Second, patients underwent various injection protocols, with various total contrast doses injected to explore a variety of bowel segment. Jacobsen et al.^25^ used phantoms to assess the limit values of detection and quantification of iodine concentrations using several DECTs on the Iqon Spectral CT. They concluded that the limit of quantification varied from 0.5 to 1 mgI/mL and the detection limit from 0.215 to 0.519 mgI/mL. In our study, the mean left colon and rectum iodine concentration was < 1 mgI/mL when the injected dose was 400 mgI/kg and 500 mgI/kg injection (regardless of the contrast agent iodine strength). Notably, the iodine concentration in the colon wall was significantly lower than in the upper bowel. Consequently, the inter-rater agreement for analyzing bowel wall iodine concentration was lower in the colon, in line with previous studies^13^. We also observed that the higher the dose of iodine injected, the higher the interobserver agreement. Therefore, we believe that in cases of suspected acute bowel disease, e.g., acute mesenteric ischemia, a 600 mgI/kg dose should be injected to ensure a correct analysis colon wall and improve the overall inter-rater agreement.

Third, we aimed to standardize the BWIC to account for inter-protocol and inter-subject variations. Since the most common reference for normalization is the aorta, we used the aortic iodine concentration to normalize the BWIC. We also used the background IC of abdominal organs. Interestingly, while the influence of the contrast agent and injection protocol characteristics disappeared after normalization with the IC of the abdominal organs, it was not the case after normalization with the aorta. Moreover, the interclass correlation coefficient between the BWIC and the IC of the background abdominal organs was higher than with the IC of the aorta. Furthermore, in pathological conditions, the normalization of IC based on the aorta may not be suitable due to variations in cardiac function and heart rate. One may argue that the liver alone may be suitable for IC normalization, but the liver IC may be influenced by numerous factors, including steatosis^26^. Consequently, our results suggest a benefit of normalizing the BWIC with the IC of the upper abdominal organs.

We acknowledge some limitations of this study. First, it was a retrospective, single-center study using a single, dual-energy CT platform, which limits the generalization of the results. However, Harsaker et al.^27^ showed in phantoms that the measured iodine concentrations were close to the nominal concentrations within the clinical range for both vendors studied (GE and Siemens). Furthermore, we included consecutive patients with normal bowel walls, limiting selection bias. Second, the values of iodine concentration were studied with only two contrast agents. However, we showed that the BWIC was independent from the contrast agent and total dose injected when properly normalized. Finally, the measurement of BWIC warrants further evaluation of pathological conditions, especially when bowel wall is thin or thickened. We consider the current study an essential first step toward defining bowel enhancement anomalies.

## Conclusion

The normal bowel wall iodine concentration decreases progressively from the stomach toward the rectum. Using normalization with a background iodine concentration of abdominal organs, this quantitative parameter is independent from the patients and contrast agent characteristics. This opens promising perspectives for the quantitative definition of bowel wall abnormalities.

## Data Availability

The datasets generated or analyzed during the study are available from the corresponding author on reasonable request.

## Abbreviations

ABI: Acute bowel ischemia
BMI: Body mass index
BWIC: Bowel wall iodine concentration
IC: Iodine concentration
LBW: Lean body weight
CT: Computed tomography
DECT: Dual-energy computed tomography
ROI: Region of interest
NIC: Normalized iodine concentration
