# Author Correction: Quantifying iodine concentration in the normal bowel wall using dual-energy CT: influence of patient and contrast characteristics

**DOI:** 10.1038/s41598-024-56763-2

**Published:** 2024-03-13

**Authors:** Majida Nehnahi, Gabriel Simon, Romain Moinet, Gael Piton, Camille Camelin, Maxime Ronot, Éric Delabrousse, Paul Calame

**Affiliations:** 1 Department of Radiology, University of Bourgogne Franche-Comté, CHU Besançon, 25030 Besançon, France; 2 Medical Intensive Care Unit, University of Bourgogne Franche-Comté, CHU Besançon, 25030 Besançon, France; 3 Department of Radiology, University Hospitals Paris Nord Val-de-Seine, AP-HP, Beaujon, 92110 Clichy, France; 4 EA 4662 Nanomedicine Lab, Imagery and Therapeutics, University of Franche-Comté, Besançon, France

Correction to: *Scientific Reports* 10.1038/s41598-023-50238-6, published online 19 December 2023

The original version of this Article contained errors in the Material and methods section.

Under the subheading, ‘Abdominal spectral CT protocol’,

“CT scans were acquired in helical multi-energy mode with a dual-layer detector allowing dual-energy and 120 kV single-energy images in a single acquisition (IQon Spectral CT, Philips Healthcare, Cleveland, OH, USA). CT scan settings were set with tube voltage between 80 and 140 kV energy every 0.5 ms, detector configuration = 64 × 0.625 mm, rotation time 0.4 s, dose modulation 14, 1.2 pitch. Unenhanced CT images were acquired in 169 patients.”

now reads:

“CT scans were acquired in helical mode with a dual-layer detector allowing conventional and dual-energy images in a single acquisition with a single kVp (IQon Spectral CT, Philips Healthcare, Cleveland, OH, USA). CT scan settings were set with tube voltage 120 kV detector configuration = 64 × 0.625 mm, rotation time 0.4 s, 1.2 pitch and dose modulation with DoseRight Index 14.”

The original Article has been corrected.

